# Selection of safe artemisinin derivatives using a machine learning-based cardiotoxicity platform and in vitro and in vivo validation

**DOI:** 10.1007/s00204-021-03058-4

**Published:** 2021-05-22

**Authors:** Onat Kadioglu, Sabine M. Klauck, Edmond Fleischer, Letian Shan, Thomas Efferth

**Affiliations:** 1 Department of Pharmaceutical Biology, Institute of Pharmaceutical and Biomedical Sciences, Johannes Gutenberg University, Staudinger Weg 5, 55128 Mainz, Germany; 2 Division of Cancer Genome Research, German Cancer Research Center (DKFZ), German Cancer Consortium (DKTK), National Center for Tumor Diseases (NCT), Heidelberg, Germany; 3 Fischer Organics GmbH, Weiler, Germany; 4 The First Affiliated Hospital, Zhejiang Chinese Medical University, Hangzhou, China

**Keywords:** Artificial intelligence, Cardiotoxicity, Drug discovery, Machine learning

## Abstract

The majority of drug candidates fails the approval phase due to unwanted toxicities and side effects. Establishment of an effective toxicity prediction platform is of utmost importance, to increase the efficiency of the drug discovery process. For this purpose, we developed a toxicity prediction platform with machine-learning strategies. Cardiotoxicity prediction was performed by establishing a model with five parameters (arrhythmia, cardiac failure, heart block, hypertension, myocardial infarction) and additional toxicity predictions such as hepatotoxicity, reproductive toxicity, mutagenicity, and tumorigenicity are performed by using Data Warrior and Pro-Tox-II software. As a case study, we selected artemisinin derivatives to evaluate the platform and to provide a list of safe artemisinin derivatives. Artemisinin from *Artemisia annua* was described first as an anti-malarial compound and later its anticancer properties were discovered. Here, random forest feature selection algorithm was used for the establishment of cardiotoxicity models. High AUC scores above 0.830 were achieved for all five cardiotoxicity indications. Using a chemical library of 374 artemisinin derivatives as a case study, 7 compounds (deoxydihydro-artemisinin, 3-hydroxy-deoxy-dihydroartemisinin, 3-desoxy-dihydroartemisinin, dihydroartemisinin-furano acetate-d3, deoxyartemisinin, artemisinin G, artemisinin B) passed the toxicity filtering process for hepatotoxicity, mutagenicity, tumorigenicity, and reproductive toxicity in addition to cardiotoxicity. Experimental validation with the cardiomyocyte cell line AC16 supported the findings from the in silico cardiotoxicity model predictions. Transcriptomic profiling of AC16 cells upon artemisinin B treatment revealed a similar gene expression profile as that of the control compound, dexrazoxane. In vivo experiments with a Zebrafish model further substantiated the in silico and in vitro data, as only slight cardiotoxicity in picomolar range was observed. In conclusion, our machine-learning approach combined with in vitro and in vivo experimentation represents a suitable method to predict cardiotoxicity of drug candidates.

**Supplementary Information:**

The online version contains supplementary material available at 10.1007/s00204-021-03058-4.

## Introduction

The drug discovery process comprises numerous and extensive preclinical analyses including in silico, in vitro, and in vivo experiments, before clinical trials with human subjects can be initiated. A main reason, why compounds do not reach clinical phases is the occurrence of non-tolerable toxicities. Besides hepatotoxicity, hematotoxicity, tumorigenicity and cardiotoxicity may pose severe and even life-threatening side effects (Cheng et al. 2017; Lee 2003; McGowan et al. 2017; Oliveira et al. 2007). Most drug candidates drop out from the development pipeline because of these toxicities. One important criterion for the screening of novel drug candidates is the absence of cardiotoxic effects (Lee et al. 2019). The selection of potentially active compounds from large databases requires in silico methods as rapid and cost-effective pre-selection step for subsequent in vitro experiments. Coupling suitable in silico analyses with toxicity predictions increase the efficiency of preclinical drug development (Briggs et al. 2012; Issa et al. 2017). The selection of both active and safe compounds represents a most critical step. With emerging new concepts in artificial intelligence and machine learning, the prediction of toxicity may become not only more time- and cost-effective but also more reasonable and reliable.

Many natural compounds and derivatives thereof have proven their effectiveness in drug therapy during the past decades. About 60% of the clinically established drugs are indeed in one way or another of natural origin (Newman and Cragg 2007). Available natural compound databases serve as an invaluable source to identify novel compounds that could possess activity against certain diseases or disorders by focusing on particular target proteins.

The sesquiterpenoid artemisinin was initially described as anti-malarial compound isolated from the plant *Artemisia annua* L. Later on, it became apparent that artemisinin and its derivatives are also active against cancer, viral infections, schistosomiasis and trypanosomiasis (Efferth 2017a, b; Efferth et al. 2002, 2008; Michaelsen et al. 2015; Mu and Wang 2018; Ooko et al. 2015; Shi et al. 2018). Hence, this class of compounds seems to be suited for further drug development. This also implies that toxicity evaluation of artemisinin derivatives is a mandatory critical step for the development of second-generation artemisinin derivatives.

In the present study, we developed a cardiotoxicity prediction model focusing on five of the most important parameters (arrhythmia, cardiac failure, heart block, hypertension, myocardial infarction). In addition, hepatotoxicity, mutagenicity, tumorigenicity, and reproductive toxicity were also evaluated with corresponding in silico tools. With these computational tools at hand, we selected a chemical library of 374 artemisinin derivatives to evaluate the toxicity and provide a panel of presumably safe artemisinin derivatives. The in silico results for these compounds have been verified in vitro with a cytotoxicity assay using human cardiomyocytes, microarray to analyze gene expression profiles and in vivo cardiotoxicity assessment on a zebrafish model.

## Material and methods

### Preparation of a library of artemisinin derivatives and calculation of chemical descriptors

The chemical structures of 374 artemisinin derivatives were retrieved from PubMed and PubChem. The Data Warrior software was used to calculate the chemical descriptors for cardiotoxicity in addition to mutagenicity, tumorigenicity, reproductive toxicity (Lopez-Lopez et al. 2019; Sander et al. 2015). After chemical descriptor calculations, correlation coefficients were calculated with SPSS software (IBM, USA). Descriptors having pairwise correlation with the cardiotoxicity parameters above 0.1 were considered to predict drug safety. For descriptors having a pairwise correlation coefficient higher than 0.9, the one with lower correlation coefficient with the cardiotoxicity parameters were excluded (Cai et al. 2018). By this strategy, relevant descriptors without the potential issue of over-fitting issue were selected to build the model.

### Cardiotoxicity prediction model establishment

**Fig.1:**
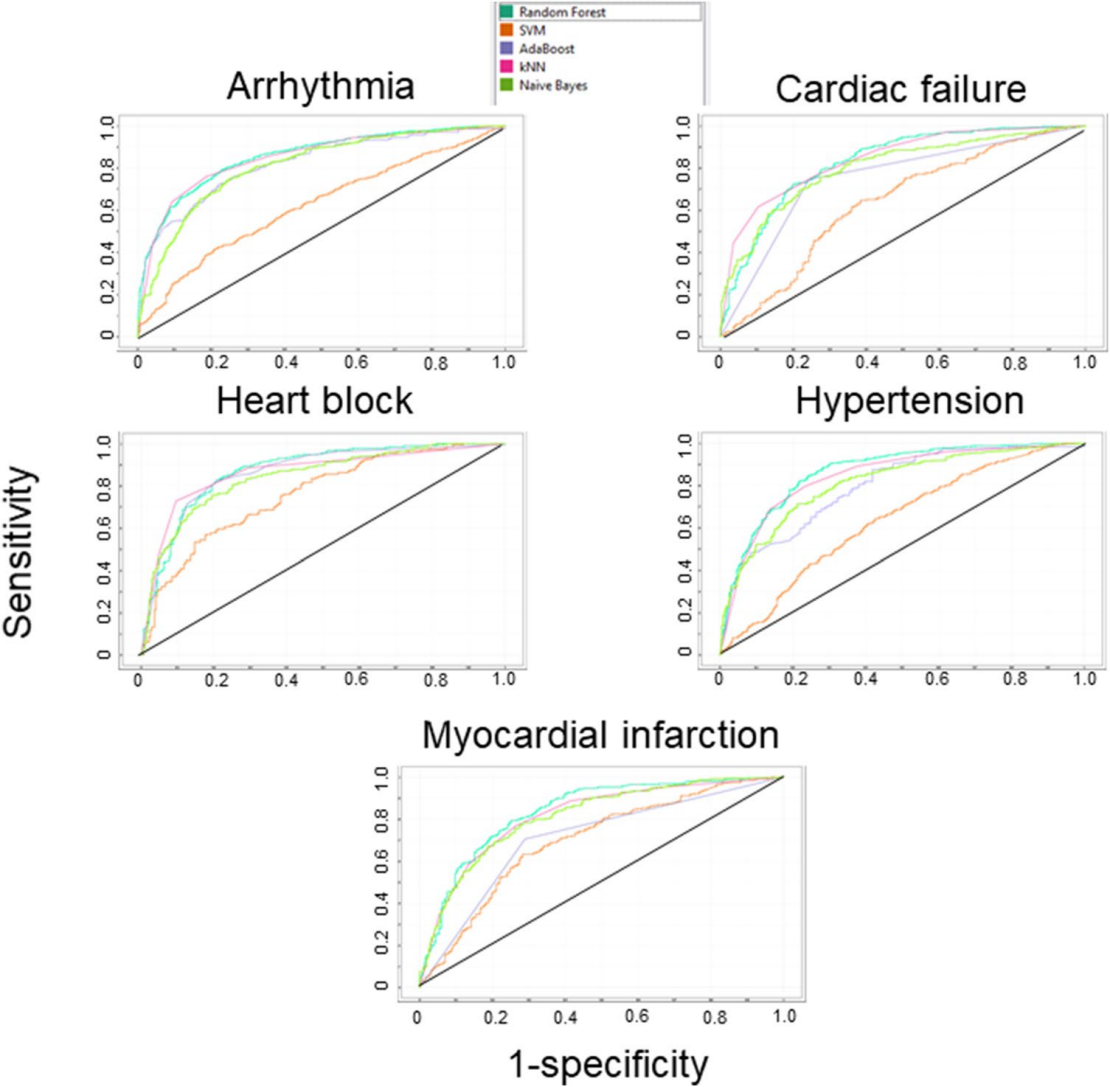
Receiver operating characteristic (ROC) curves of Ada Boost, kNN, Naive Bayes, RF, SVM classification algorithms based on leave-one-out sampling for cardiotoxicity assessment models

The establishment of a prediction model for cardiotoxicity was performed by using the machine-learning software Orange (Ljubljana, Slovenia) (Demsar et al. 2013; Kadioglu and Efferth 2019) and the cardiotoxicity training subset of compounds deposited in the DrugBank database (https://www.drugbank.ca/). A total of 1451 compounds for arrhythmia, 626 compounds for cardiac failure, 545 compounds for heart block, 1163 compounds for hypertension, and 639 compounds for myocardial infarction were selected for the learning steps and model establishment. For further external validation and evaluation of the models, additional 143 compounds for arrhythmia, 538 compounds for cardiac failure, 403 compounds for heart block, 291 compounds for hypertension, and 179 compounds for myocardial infarction were selected. After applying the descriptor selection criteria by considering appropriate relevance and over-fitting issues, “logP”, “drug likeness”, “amines”, “ligand efficiency (LE)”, “alkyl-amines”, “aromatic nitrogens” and “basic nitrogens” were considered for model preparation. Various classification algorithms with the “leave-one-out” sampling method were tested, i.e. Ada Boost, k-nearest neighboring (kNN), Naive Bayes, random forest (RF), and support vector machine (SVM). Receiver operating characteristic (ROC) curves are depicted in Fig. 1. Based on this training set of known cardiotoxic compounds, the further evaluation of the established cardiotoxicity models was performed by using a list taken from the literature (Mladenka et al. 2018). The RF algorithm performed better than the other classification algorithms. Overall performance for the established models is summarized in Table 1.

**Table 1:** Performance of the in silico cardiotoxicity models based on the random forest classifier algorithm

Cardiotoxicity models	Machine learning					External validation set			
	AUC	sensitivity	specificity	overall predictive accuracy	precision	sensitivity	specificity	overall predictive accuracy	precision
Arrhythmia	0.849	0.775	0.764	0.770	0.767	0.887	0.789	0.838	0.808
Cardiac failure	0.831	0.768	0.742	0.755	0.752	0.804	0.730	0.767	0.751
Heart block	0.869	0.783	0.779	0.781	0.780	0.881	0.806	0.843	0.819
Hypertension	0.854	0.795	0.766	0.781	0.773	0.862	0.676	0.769	0.727
Myocardial infarction	0.834	0.765	0.759	0.762	0.760	0.820	0.753	0.787	0.768

### Prediction of other toxicities

The prediction of hepatotoxicity and immunotoxicity was performed with the Pro-Tox-II web server (Banerjee et al. 2018). Additional toxicity parameters (mutagenicity, tumorigenicity, reproductive toxicity) were evaluated with Data Warrior software.

### Verification of cardiotoxicity predictions

At first, molecular docking on hERG (human ether-a-go-go related gene, alias *KCNH2*) protein (PDB ID: 5VA2) was performed to assess the interaction between the selected compounds and the well-known cardiotoxicity marker protein hERG. Doxorubicin is a well-known cardiotoxic drug, which was used as positive control for cardiotoxicity. Dexrazoxane is a safe cardiovascular agent, which has protective effects towards cardiotoxicity caused by anthracyclines (Liesse et al. 2018; Narayan et al. 2019; Zhang et al. 2015). This drug was utilized as negative control for cardiotoxicity. Briefly, the AutoDock 4 algorithm (Morris et al. 2009) was used for the molecular docking calculations with 2.500.000 energy evaluations and 250 runs. The AutoDock Tools 1.5.7 software (Morris et al. 2009) was applied for visualization and preparation of ligand and protein structures. The artemisinin compounds selected by the machine learning-based RF algorithm described above have been subjected to an automated and comprising molecular docking campaign by using the high-performance supercomputer MOGON (Johannes Gutenberg University, Mainz). Compound flexibilities were taken into account and a rigid receptor structure was used. The binding site of hERG was retrieved from the literature (Wang and MacKinnon 2017). Three independent defined docking runs were performed by covering the binding site on hERG. Weak binding with hERG implies safe drug profiles (Bhat and Houghton 2018; Lee et al. 2019).

### Cytotoxicity assay

To verify the cardiotoxicity of the selected compounds further, the cardiomyocyte cell line AC16 was used, which was kindly provided by Dr. James Spiers (Trinity College Dublin, Department of Pharmacology and Therapeutics). It was cultured as previously described (Elgenaidi and Spiers 2019). The cytotoxicity of the selected compounds (provided by one of the authors, E.F.) and of doxorubicin (provided by University of Mainz Clinical Pharmacy as positive control drug) towards AC16 cardiomyocytes was evaluated by the resazurin assay with three independent experiments per compounds and each 6 parallel measurements per experiment as previously described (Ooko et al. 2016).

### Microarray analyses

Microarray experiments for artemisinin B, doxorubicin and dexrazoxane were performed with 10 µM concentration for artemisinin B and dexrazoxane on AC16 cells for 24 h, whereas the IC50 concentration was used for doxorubicin (1.918 µM). RNA extraction and quality control were performed as previously described (Yan et al. 2020). Microarray analyses using the Affymetrix Clariom S assay (Affymetrix, Santa Clara, CA, USA) chips according to the manufacturer protocol were conducted by the Genomics and Proteomics Core Facility at the German Cancer Research Center (DKFZ, Heidelberg). Statistical analysis was performed with the Chipster software (Helsinki, Finland).

### Zebrafish handling and acute toxicity assay

Wild-type AB strain of adult zebrafish was purchased from the China Zebrafish Resource Center, Institute of Hydrobiology, China Academy of Science (Wuhan, China) and accredited by the Association for Assessment and Accreditation of Laboratory Animal Care International (SYXK 2012–0171). Zebrafish larvae 48 h post fertilization (hpf) were obtained by natural pair-mating and housed in a light-controlled aquaculture facility with a standard 14:10 h day/night photoperiod and fed with live brine shrimp twice a day and fry flakes once a day.

Totally, 210 zebrafish larvae 48 hpf were employed and divided into 7 groups in 6-well plates (Nest Biotech, China) with 30 fishes in each group. The basic procedure of zebrafish handling and drug treatment was recently described by us (Zheng et al. 2020). Artemisinin B was dissolved with DMSO (Sigma Aldrich, Germany) for treatment groups, and DMSO only was used for DMSO group. Artemisinin B at 0, 0.0625, 0.125, 0.25, 0.5, and 1.0 pg/ml were respectively microinjected into the yolk sac of each zebrafish larvae. All fishes were subject to visual observation and image acquisition under a dissecting stereomicroscope (Olympus Ltd, Tokyo, Japan). The death number and adverse event of zebrafishes were recorded for each group.

## Results

### Toxicity predictions

We developed a machine learning-based toxicity prediction platform focusing on cardiotoxicity, hepatotoxicity, immunotoxicity, mutagenicity, reproductive toxicity and tumorigenicity. First, we generated a cardiotoxicity prediction model with a leave-one-out sampling based random forest algorithm for five main parameters of cardiotoxicity (arrhythmia, cardiac failure, heart block, hypertension, myocardial infarction) with high-performance scores for both learning and external validation sets. The sensitivity for the learning set was in the range 0.765 to 0.795 and for the external validation set between 0.804 and 0.887 (Table 1). The specificity, overall predictive accuracy and precision for the learning and external validation set ranged between 0.676 and 0.843 (Table 1).

**Fig.2:**
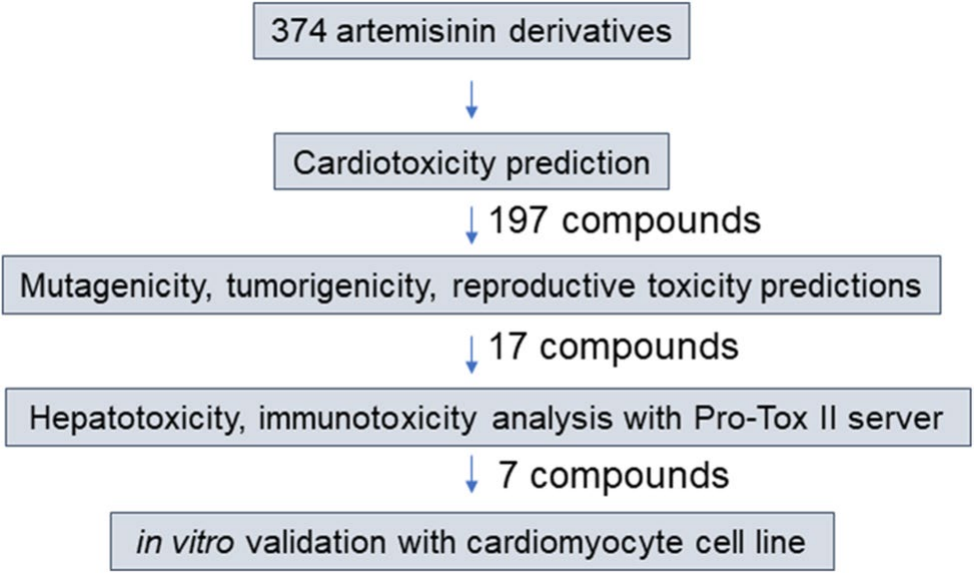
Computational filtering steps to select safe artemisinin derivatives

Secondly, we coupled the cardiotoxicity platform with software for predicting hepatotoxicity, immunotoxicity, mutagenicity, reproductive toxicity and tumorigenicity, in order to provide a sufficient filtering method to select safe compounds from any given compound library. A set of 374 artemisinin derivatives, which was retrieved from PubMed (Supplementary Table 1), was used as exemplary case study in the present investigation. Overall, the main aim to use the artemisinin derivatives is to provide novel non-toxic artemisinin derivatives for further drug development. Step-by-step filtering and scheme for the selection of safe compounds are depicted in Fig. 2. The first round of filtering with our cardiotoxicity model delivered 197 compounds (~ 53%) (Supplementary Table 2) as potentially safe with low cardiotoxicity features. As a next filtering step, mutagenicity, tumorigenicity and reproductive toxicity were assessed by using DataWarrior software. Here, 17 compounds out of 197 were selected (Supplementary Table 3). The last filtering step was performed by using the Pro-Tox II software focusing on hepatotoxicity and immunotoxicity, and finally resulted in 7 out of 17 compounds (deoxydihydro-artemisinin, 3-hydroxy-deoxy-dihydroartemisinin, 3-desoxy-dihydroartemisinin, dihydroartemisinin-furano acetate-d3, deoxyartemisinin, artemisinin G, artemisinin B).

A widely accepted marker to evaluate the interaction of chemical compounds with cardiotoxicity is hERG (Bhat and Houghton 2018; Luo et al. 2014).

As a third step in the prediction process, we chose to perform an in silico evaluation of the models for cardiotoxicity and the additional parameters (mutagenicity, tumorigenicity, reproductive effect, immunotoxicity and hepatotoxicity) with a list of 39 compounds with known cardiotoxicity taken from the literature (Mladenka et al. 2018). Here, a 97.4% success rate was achieved, as 38 of them were predicted to involve toxicity. Thirty-four compounds were successfully predicted as cardiotoxic (Table 2), and the next step of filtering with the additional parameters led to the prediction of four more compounds as toxic (malathion: mutagenicity, tumorigenicity, reproductive toxicity; methyldopa: reproductive toxicity; grayanotoxin III 6,14 diacetate: immunotoxicity; sorafenib: immunotoxicity and hepatotoxicity).

These results made us confident to select the remaining set of 7 compounds of artemisinin derivatives from the machine learning-based toxicity prediction platform for further experimental in vitro and in vivo validation.

**Table 2:** Performance of the in silico cardiotoxicity prediction

Name	Predicted cardiotoxicity	Name	Predicted cardiotoxicity
5-Fluorouracil	Yes	Lidocain	Yes
Aconitine	Yes	Malathion*	No
Amifostine	Yes	Metamphetamine	Yes
Amphetamine	Yes	Methyldopa*	No
Atropine	Yes	Methyltestosterone	Yes
Chloroprocaine	Yes	Metoprolol	Yes
Clenbuterol	Yes	Milrinone	No
Clonidine	Yes	Nortrpytiline	Yes
Cocaine	Yes	Paclitaxel	Yes
Digoxin	Yes	Phenylephrine	Yes
Dobutamine	Yes	Prednisone	Yes
Doxazosin	Yes	Reserpine	Yes
Doxorubicin	Yes	Ritodrine	Yes
Grayanotoxin iii 6,14 diacetate*	Yes	Rofecoxib	Yes
Ibuprofen	Yes	Salbutamol	Yes
Ibutilide	Yes	Sildenafil	Yes
Isoprenaline	Yes	Sorafenib*	No
Lapatinib	Yes	Theohylline	Yes
Levosimendan	Yes	Verapamil	Yes
		Yohimbine	Yes

Compounds labeled with * exert mutagenicity, tumorigenicity, reproductive effect, immunotoxicity or hepatotoxicity

### Verification of cardiotoxicity in silico predictions

A widely accepted marker to evaluate the interaction of chemical compounds with cardiotoxicity is hERG (Bhat and Houghton 2018; Luo et al. 2014). Provided that a compound has a weak interaction with hERG, a safe profile in terms of cardiotoxicity can be assumed (Bhat and Houghton 2018; Lee et al. 2019). As a first step, the interactions of the investigational compounds with hERG were evaluated by means of molecular docking. All compounds except deoxyartemisinin and artemisinin G revealed weaker binding than doxorubicin (Table 3), which is a well-known cardiotoxic drug, also being labeled cardiotoxic in the in silico prediction testing (Table 2). Dihydroartemisinin furano acetate-d3 and artemisinin B revealed the weakest interaction with hERG, which can be taken as a clue for missing cardiotoxicity. Remarkably, artemisinin B possessed even weaker binding than the cardioprotective control drug, dexrazoxane (Table 3).

**Table 3:** Molecular docking results of selected artemisinin derivatives on hERG

Compound	LBE (kcal/mol)	Predicted inhibition constant (µM)
Deoxydihydro-artemisinin	− 5.050 ± 0.000	198.777 ± 0.235
3-Hydroxydeoxy-dihydroartemisinin	− 5.053 ± 0.006	197.417 ± 0.556
3-Desoxy-dihydroartemisinin	− 5.050 ± 0.000	200.210 ± 0.113
Dihydroartemisinin-furanoacetate	− 4.893 ± 0.006	258.980 ± 1.825
Deoxyartemisinin	− 5.230 ± 0.000	146.603 ± 0.391
Artemisinin G	− 5.200 ± 0.000	155.018 ± 0.295
Artemisinin B	− 4.467 ± 0.031	531.677 ± 29.573
Doxorubicin (positive control)	− 5.160 ± 0.066	166.230 ± 17.840
Dexrazoxane (negative control)	− 4.570 ± 0.000	449.210 ± 1.417

**Fig.3:**
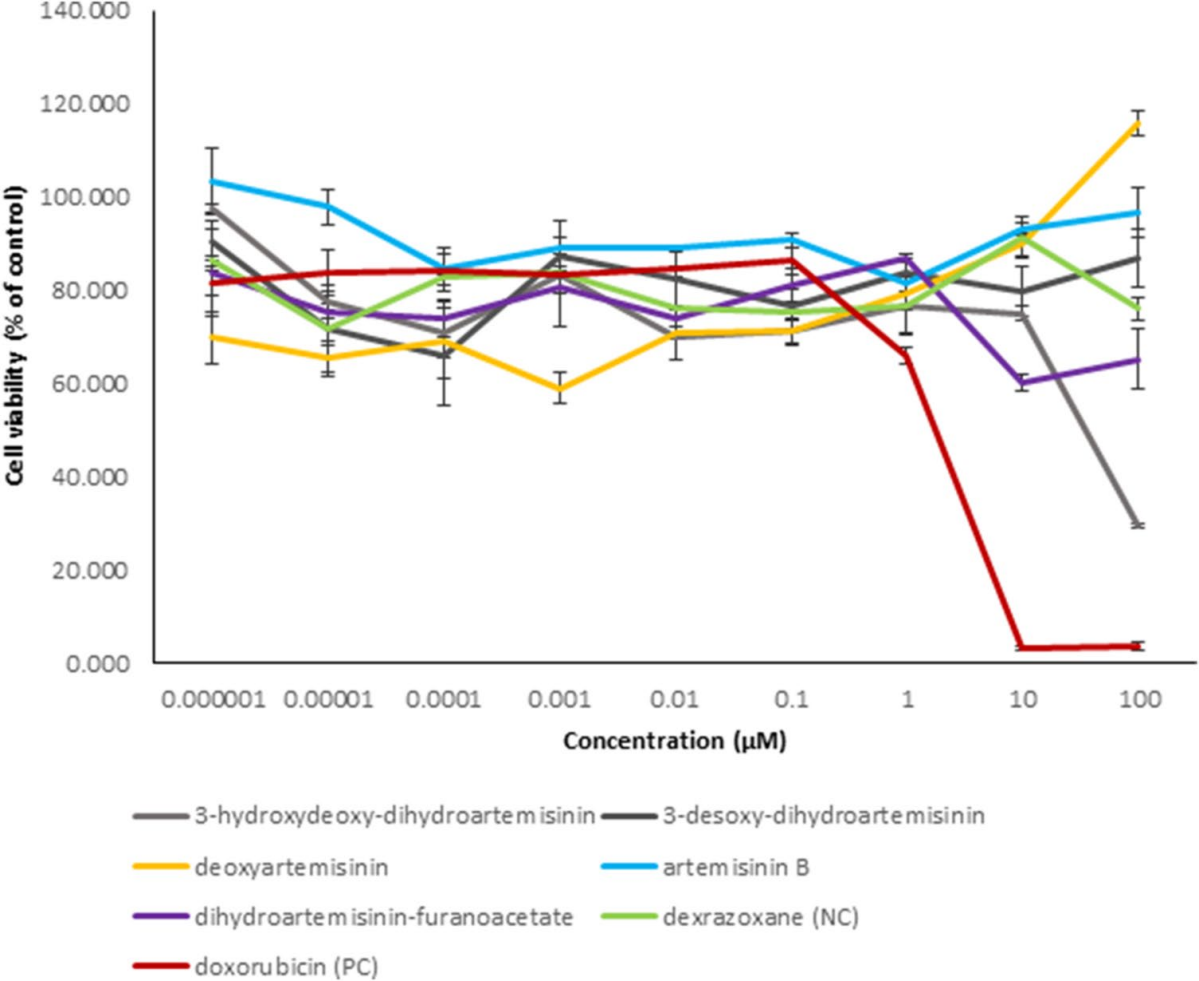
Cytotoxicity assessment of the selected compounds on AC16 cardiomyocytes. Results represent mean values and standard deviation of each three independent experiments with each 6 parallel measurements

Five out of the 7 selected compounds were available to us to investigate cytotoxicity in vitro towards AC16 cardiomyocytes as a parameter to measure cellular cardiotoxicity. As shown in Fig. 3, none of the selected compounds revealed cytotoxicity. The percentages of cell viabilities were quite comparable to those of the cardioprotective control compound, dexrazoxane, except of 3-hydroxydeoxy-dihydroartemisinin. Here, treatment with a high concentration of 100 µM revealed a moderate cytotoxic effect (IC_50_: 24.915 ± 0.247 µM). Considerable cytotoxicity towards AC16 cardiomyocytes was solely observed with doxorubicin. Here, the IC_50_ value was 1.918 ± 0.230 µM. It was not possible to measure IC_50_ values for all other artemisinin derivatives as well as dexrazoxane in concentrations up to 100 µM. Even cell viability at high concentrations for artemisinin B and deoxyartemisinin was similar to cytotoxicity results previously reported for spinochrome D on AC16 cell line (Yoon et al. 2018). Overall, these results indicate that the six artemisinin derivatives may reveal a sufficient cardiosafety profile for their use in vivo.

### Toxicity evaluation of artemisinin B

**Fig.4:**
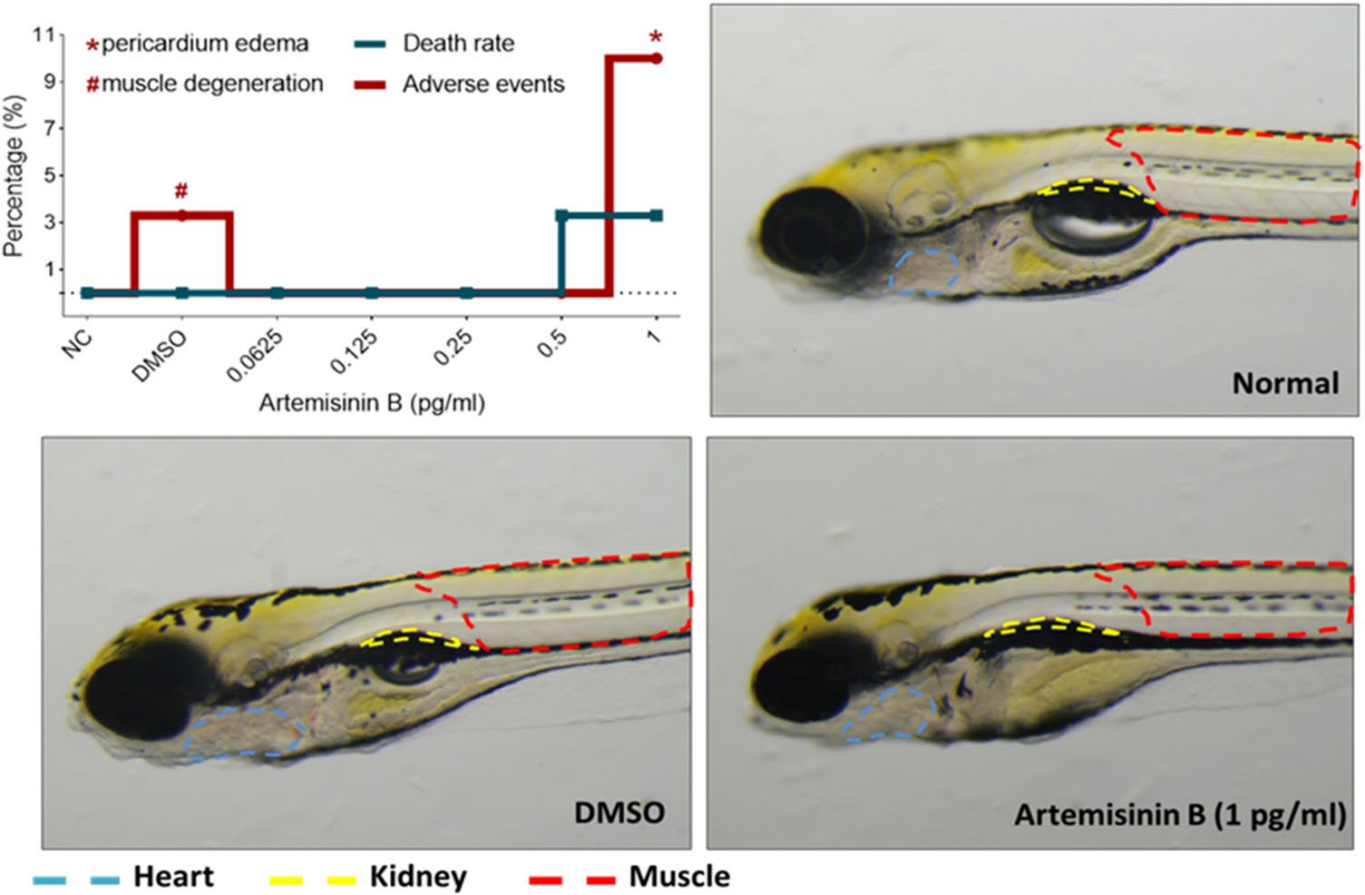
Mortality and adverse events of zebrafish larvae upon artemisinin B treatment

The compound artemisinin B with the best performance in the molecular docking and cell viability experiments was finally tested in the Zebrafish model. The curves of death rate and adverse events of zebrafish larvae are shown in Fig. 4. Zebrafish death was observed with artemisinin B at 0.5 pg/ml, and only one dead fish (3.3% death rate) was found. A death rate below 10% can be regarded as experimental error. Pericardial edema was observed as the only possible adverse event induced by artemisinin B at 1.0 pg/ml. Since the incidence rate of adverse event was less than 10% which can be accepted as normal response, 1.0 pg/ml can still be estimated as safe dose of artemisinin B. Therefore, artemisinin B at a dose range within 1.0 pg/ml was non-toxic to zebrafish larvae.

### Transcriptomic analyses

Transcriptome-wide RNA expression analyses revealed that artemisinin B treatment of AC16 cardiomyocytes led to deregulations in cardiotoxicity marker genes in a similar manner as dexrazoxane, and it caused the upregulation of various cardiotoxicity marker genes, which are known to be associated with doxorubicin associated cardiotoxicity. Referring to the literature, where potential cardiotoxicity marker genes were reported (Yoon et al. 2018) and considering those genes, *APP* was 644-fold upregulated, *CCND1* was 385-fold upregulated, *NT5E* was 774-fold upregulated, *PCNA* was 126-fold upregulated, *PRNP* was 360-fold upregulated, *STK39* was 60-fold upregulated, *TXNIP* was 371-fold upregulated by artemisinin B.

*AIFM1* was 170-fold downregulated, *APP* was 633-fold downregulated, *ARAF* was 675-fold downregulated, *CBR1* was 650-fold downregulated, *CNP* was 160-fold downregulated, *DLD* was 394-fold downregulated, *GSK3B* was 697-fold downregulated, *NBN* was 189-fold downregulated, *NT5E* was 510-fold downregulated, *PBX3* was 222-fold downregulated, *RAB2A* was 788-fold downregulated, *SP110* was 264-fold downregulated, *UBE4B* was 540-fold downregulated, *VIM* was 558-fold downregulated by doxorubicin.

*CTSC* was 291-fold upregulated, *DVL1* was 66-fold upregulated, *KLF6* was 163-fold upregulated, *MUS81* was 50-fold upregulated, *NT5E* was 603-fold upregulated, *PFN1* was 562-fold upregulated, *PRNP* was 283-fold upregulated by dexrazoxane (Table 4).

**Table 4:** Fold change of cardiotoxicity marker genes in artemisinin B-, dexrazoxane- and doxorubicin-treated AC16 cells

Gene	Artemisinin B	Gene	Dexrazoxane	Gene	Doxorubicin
*APP*	644.150	*CTSC*	291.693	*AIFM1*	− 170.564
*CCND1*	385.042	*DVL1*	66.551	*APP*	− 633.831
*NT5E*	774.197	*KLF6*	163.719	*ARAF*	− 675.091
*PCNA*	126.363	*MUS81*	50.017	*CBR1*	− 650.829
*PRNP*	360.351	*NT5E*	603.894	*CNP*	− 160.800
*STK39*	60.882	*PFN1*	562.100	*DLD*	− 394.123
*TXNIP*	371.243	*PRNP*	283.204	*GSK3B*	− 697.101
				*NBN*	− 189.766
				*NT5E*	− 510.647
				*PBX3*	− 222.206
				*RAB2A*	− 788.420
				*SP110*	− 264.249
				*UBE4B*	− 540.823
				*VIM*	− 558.631

Seventy-eight genes were commonly deregulated in artemisinin B and dexrazoxane and 67 of them are both down- or upregulated in artemisinin B and dexrazoxane-treated cells (Supplementary Table 4). Twenty-eight genes are commonly deregulated in all treatments and 9 out of those 28 genes are reversely deregulated in artemisinin B and dexrazoxane-treated cells compared to doxorubicin-treated cells (Table 5), implying cardiotoxicity features of these genes (*ANPEP*, *BACE1*, *C1QBP*, *MGLL*, *MT1A*, *MT1B*, *NT5E*, *RGS4*, *RPL23A*).

**Table 5:** Fold change of reversely deregulated genes in artemisinin B- or dexrazoxane-treated AC16 cells compared to doxorubicin-treated AC16 cells.

Gene	Artemisinin B	Dexrazoxane	Doxorubicin
*ANPEP*	503.888	154.716	− 1166.090
*BACE1*	− 60.065	− 38.736	131.002
*C1QBP*	251.246	296.638	− 1196.785
*MGLL*	41.710	205.947	− 229.316
*MT1A*	3050.855	2360.951	− 2028.899
*MT1B*	1895.162	1362.674	− 1498.303
*NT5E*	774.197	603.894	− 510.647
*RGS4*	256.294	348.026	− 361.650
*RPL23A*	− 611.567	− 612.719	676.744

## Discussion

Recent advances of in silico approaches and tools are promising in terms of systematic evaluation of drug-induced cardiovascular (CV) complications in both drug discovery and post-marketing surveillance (Collins et al. 2015; Lorberbaum et al. 2016a, 2016b). Recently, a machine learning-based cardiotoxicity prediction platform has been reported (Cai et al. 2018). In the present study, we generated models based on random forest algorithm with leave-one-out cross-validation to predict cardiotoxicities of different categories in a similar manner as described by Cai et al. (2018). Moreover, we considered other toxicities as well with the use of additional software applications. Our models outperformed in all categories in learning steps except for specificity (0.779 vs. 0.813) and precision (0.780 vs. 0.794) of the heart block model and specificity (0.759 vs. 0.765) of the myocardial infarction model. Regarding the external validation set, we outperformed all categories except for specificity (0.676 vs. 0.710) of the hypertension model. Seven compounds of artemisinin derivatives (deoxydihydro-artemisinin, 3-hydroxy-deoxy-dihydroartemisinin, 3-desoxy-dihydroartemisinin, dihydroartemisinin-furano acetate-d3, deoxyartemisinin, artemisinin G, artemisinin B) passed the toxicity filtering process not only as non-cardiotoxic compounds but also as safe compounds in terms of hepatotoxicity, mutagenicity, tumorigenicity, and reproductive toxicity by including those additional toxicity parameters. Even if drug candidates exert promising pharmacological features towards their therapeutic disease-specific target, non-tolerable toxicities may lead to a drop out of these compounds from the drug development pipeline. For this purpose, suitable toxicity profiling of candidate drugs is essential. The presented toxicity prediction platform was highly accurate and reliable and may, therefore, offer the opportunity to speed up the preclinical drug discovery process and to reduce the developmental costs. The platform proved its success rate in external validation sets, and selected artemisinin derivatives revealed weak interaction with hERG and a comparable safety profile with that of a cardioprotective agent, dexrazoxane, on cardiomyocytes.

Drug attrition and thus approval failure in the last 20 years were mainly due to the lack of efficacy (accounting for ~ 30% of failures) and safety (toxicology and clinical safety accounting for a further ~ 30% (Ferri et al. 2013)). Cardiovascular toxicity is among the most frequent serious adverse drug reactions and cause of withdrawal for marketed drugs. Therefore, it is of utmost importance to identify and characterize the cardiotoxicity risk of drug candidates as early as possible at the preclinical stage of development (Redfern et al. 2010). Safety liabilities associated with the cardiovascular system account for 45% of the total post-approval drug withdrawal from the market, compared to 32% for the hepatic system (Stevens and Baker 2009). Such a high incidence and severity of cardiovascular toxicity in the late-stage of clinical development can lead to restrictions in indications and dose levels, pre- and/or post-approval monitoring, and ultimately drug discontinuation or withdrawal (Ferri et al. 2013).

Considering the cardiotoxicity marker genes, which are deregulated in doxorubicin-treated cardiomyocytes (Yoon et al. 2018), artemisinin B treatment caused an upregulation of 7 genes (*APP*, *CCND1, NT5E*, *PCNA*, *PRNP*, *STK39*, *TXNIP*), while treatment with the cardioprotective control, dexrazoxane, revealed an upregulations of another 5 genes (*CTSC*, *DVL1*, *KLF6*, *MUS81*, *PFN1*), but interestingly also *NT5E* and *PRNP*. By contrast, doxorubicin led to the downregulation of 14 genes (*AIFM1, APP, ARAF, CBR1, CNP, DLD, GSK3B, NBN, NT5E, PBX3, RAB2A, SP110, UBE4B, VIM*).

Nine genes (including *NT5E*) were reversely deregulated in artemisinin B- or dexrazoxane-treated cells compared to doxorubicin-treated cells, implying cardiotoxicity marker properties of these genes (*ANPEP*, *BACE1*, *C1QBP*, *MGLL*, *MT1A*, *MT1B*, *NT5E*, *RGS4*, *RPL23A*) in addition to the genes mentioned above. *RGS4*-null mice showed a reduced basal heart rate (Stewart et al. 2012) and enhanced bradycardia resulting from *RGS4* loss due to intrinsic alterations in cardiac automaticity (Cifelli et al. 2008). Artemisinin B and dexrazoxane treatments led to the upregulation of *RGS4,* whereas doxorubicin downregulated *RGS4*, implying cardioprotection by artemisinin B. Among the previously reported doxorubicin-related downregulated cardiotoxicity marker genes (Todorova et al. 2012), *APP, CCND1, NT5E, PCNA, PRNP, STK39, TXNIP* were upregulated in artemisinin B-treated cardiomyocytes, further supporting the cardioprotective properties of artemisinin B.

The cardiotoxic potential of artemisinin derivatives has been reported in dogs (Yin et al. 2014). However, human trials did not show considerable heart function impairment signs by artemisinin derivatives (Efferth and Kaina 2010). This is presumably due to the low dose of artesunate applied in malarial therapy (Efferth and Kaina 2010). The therapeutically effective doses may be different for the treatment of cancer or other diseases. Therefore, the safety of artemisinins in malaria treatment does not allow to extrapolate on safety for other diseases. Only via dose escalation studies in animal and human studies, the appropriate cardiotoxic profiles of artemisinin derivatives can be reliably determined.

Radioligand binding assays, electrophysiology measurements, rubidium-flux assays, and fluorescence-based assays are among the methods to assess cardiotoxicity (Polak et al. 2009). Zebrafish modeling is another widely used strategy in that regard (Sarmah and Marrs 2016). However, such methods are less feasible for the evaluation of a large number of compounds in early-stage drug discovery because of high expenses and low throughput. After in silico filtering and in vitro experimentation, we selected artemisinin B and tested it in a zebrafish model, because artemisinin B revealed the weakest interaction with hERG and the lowest cytotoxicity towards AC16 cardiomyocytes. Our results indicate that artemisinin B at 1.0 pg/ml was non-toxic to zebrafish larvae. We conclude that artemisinin B is a promising non-cardiotoxic compound, since it revealed a similar gene expression profile to that of dexrazoxane and showed only slight cardiotoxicity in the picomolar range in zebrafish.

## Conclusions

In the present study, we established a toxicity prediction platform involving cardiotoxicity, hepatotoxicity, immunotoxicity, tumorigenicity, mutagenicity, and reproductive toxicity. Random forest feature selection algorithm was used for the establishment of cardiotoxicity models, and high AUC scores above 0.830 were achieved for all five cardiotoxicity indications. Using artemisinin derivatives as example, 7 out of 374 compounds passed the toxicity filtering procedure. Selected compounds revealed weak interaction with hERG, low toxicity towards cardiomyocytes, verifying their safe profile. Artemisinin B treatment revealed a similar gene expression profile to that of dexrazoxane and showed only slight cardiotoxicity in the picomolar range in zebrafish. In conclusion, our toxicity prediction scheme can speed up the identification of safe compounds in preclinical drug development.

## Supplementary Information

Below is the link to the electronic supplementary material.

Supplementary file1 (XLSX 33 kb)

## Data Availability

All data generated or analyzed during this study are included in this published article.

## Abbreviations

AUC: Area under the curve
hERG: Human ether-a-go-go related gene
kNN: K-nearest neighboring
RF: Random forest
ROC: Receiver operating characteristic
SVM: Support vector machine

## References

[CR1] Banerjee P, Eckert AO, Schrey AK, Preissner R (2018). ProTox-II: a webserver for the prediction of toxicity of chemicals. Nucleic Acids Res.

[CR2] Bhat R, Houghton M (2018). A human cardiomyocyte cell-line expressing hERG: an improved system for testing drug-associated hERG blocking and cardiotoxicity. J Pharmacol Tox Met.

[CR3] Briggs K, Cases M, Heard DJ (2012). Inroads to predict in vivo toxicology-an introduction to the eTOX Project. Int J Mol Sci.

[CR4] Cai C, Fang J, Guo P (2018). In silico pharmacoepidemiologic evaluation of drug-induced cardiovascular complications using combined classifiers. J Chem Inf Model.

[CR5] Cheng YJ, Wu R, Cheng ML (2017). Carboplatin-induced hematotoxicity among patients with non-small cell lung cancer: Analysis on clinical adverse events and drug-gene interactions. Oncotarget.

[CR6] Cifelli C, Rose RA, Zhang H (2008). RGS4 regulates parasympathetic signaling and heart rate control in the sinoatrial node. Circ Res.

[CR7] Collins TA, Bergenholm L, Abdulla T (2015). Modeling and simulation approaches for cardiovascular function and their role in safety assessment. Cpt-Pharmacomet Syst.

[CR8] Demsar J, Curk T, Erjavec A (2013). Orange: data mining toolbox in python. J Mach Learn Res.

[CR9] Efferth T (2017). Cancer combination therapies with artemisinin-type drugs. Biochem Pharmacol.

[CR10] Efferth T (2017). From ancient herb to modern drug: artemisia annua and artemisinin for cancer therapy. Semin Cancer Biol.

[CR11] Efferth T, Kaina B (2010). Toxicity of the antimalarial artemisinin and its dervatives. Crit Rev Toxicol.

[CR12] Efferth T, Bauer R, Funk JO, Davey M, Volm M, Davey R (2002). Molecular modes of action of antimalarial artemisinin derivatives as novel anticancer drugs. Eur J Cancer.

[CR13] Efferth T, Romero MR, Wolf DG, Stamminger T, Marin JJG, Marschall M (2008). The antiviral activities of artemisinin and artesunate. Clin Infect Dis.

[CR14] Elgenaidi IS, Spiers JP (2019). Hypoxia modulates protein phosphatase 2A through HIF-1 alpha dependent and independent mechanisms in human aortic smooth muscle cells and ventricular cardiomyocytes. Brit J Pharmacol.

[CR15] Ferri N, Siegl P, Corsini A, Herrmann J, Lerman A, Benghozi R (2013). Drug attrition during pre-clinical and clinical development: understanding and managing drug-induced cardiotoxicity. Pharmacol Ther.

[CR16] Issa NT, Wathieu H, Ojo A, Byers SW, Dakshanamurthy S (2017). Drug metabolism in preclinical drug development: a survey of the discovery process, toxicology, and computational tools. Curr Drug Metab.

[CR17] Kadioglu O, Efferth T (2019) A machine learning-based prediction platform for p-glycoprotein modulators and its validation by molecular docking. Cells. 10.3390/cells8101286 10.3390/cells8101286 PMC6829872 31640190

[CR18] Lee WM (2003). Medical progress: drug-induced hepatotoxicity. New Engl J Med.

[CR19] Lee HM, Yu MS, Kazmi SR (2019). Computational determination of hERG-related cardiotoxicity of drug candidates. BMC Bioinform.

[CR20] Liesse K, Harris J, Chan M, Schmidt ML, Chiu B (2018). Dexrazoxane significantly reduces anthracycline-induced cardiotoxicity in pediatric solid tumor patients: a systematic review. J Pediatr Hematol Oncol.

[CR21] Lopez-Lopez E, Naveja JJ, Medina-Franco JL (2019). DataWarrior: an evaluation of the open-source drug discovery tool. Expert Opin Drug Dis.

[CR22] Lorberbaum T, Sampson KJ, Chang JB (2016). Coupling data mining and laboratory experiments to discover drug interactions causing QT prolongation. J Am Coll Cardiol.

[CR23] Lorberbaum T, Sampson KJ, Woosley RL, Kass RS, Tatonetti NP (2016). An integrative data science pipeline to identify novel drug interactions that prolong the QT interval. Drug Saf.

[CR24] Luo F, Gu JY, Chen LR, Xu XJ (2014). Molecular docking and molecular dynamics studies on the structure-activity relationship of fluoroquinolone for the HERG channel. Mol Bio Syst.

[CR25] McGowan JV, Chung R, Maulik A, Piotrowska I, Walker JM, Yellon DM (2017). Anthracycline chemotherapy and cardiotoxicity. Cardiovasc Drug Ther.

[CR26] Michaelsen FW, Saeed MEM, Schwarzkopf J, Efferth T (2015). Activity of Artemisia annua and artemisinin derivatives, in prostate carcinoma. Phytomed Int J Phytotherapy Phytopharmacol.

[CR27] Mladenka P, Applova L, Patocka J (2018). Comprehensive review of cardiovascular toxicity of drugs and related agents. Med Res Rev.

[CR28] Morris GM, Huey R, Lindstrom W (2009). AutoDock4 and AutoDockTools4: automated docking with selective receptor flexibility. J Comput Chem.

[CR29] Mu XZ, Wang CC (2018) Artemisinins—a promising new treatment for systemic lupus erythematosus: a descriptive review. Curr Rheumatol Rep. 10.1007/s11926-018-0764-y 10.1007/s11926-018-0764-y 30056574

[CR30] Narayan HK, Putt ME, Kosaraju N (2019). Dexrazoxane preferentially mitigates doxorubicin cardiotoxicity in female children with sarcoma. Open Heart.

[CR31] Newman DJ, Cragg GM (2007). Natural products as sources of new drugs over the last 25 years. J Nat Prod.

[CR32] Oliveira PA, Colaco A, Chaves R, Guedes-Pinto H, De-La-Cruz LF, Lopes C (2007). Chemical carcinogenesis. An Acad Bras Cienc.

[CR33] Ooko E, Saeed MEM, Kadioglu O (2015). Artemisinin derivatives induce iron-dependent cell death (ferroptosis) in tumor cells. Phytomed In J Phytother Phytopharmacol.

[CR34] Ooko E, Alsalim T, Saeed B (2016). Modulation of P-glycoprotein activity by novel synthetic curcumin derivatives in sensitive and multidrug-resistant T-cell acute lymphoblastic leukemia cell lines. Toxicol Appl Pharm.

[CR35] Polak S, Wisniowska B, Brandys J (2009). Collation, assessment and analysis of literature in vitro data on hERG receptor blocking potency for subsequent modeling of drugs' cardiotoxic properties. J Appl Toxicol.

[CR36] Redfern W, Ewart L, Hammond T (2010). Impact and frequency of different toxicities throughout the pharmaceutical life cycle. The Toxicologist.

[CR37] Sander T, Freyss J, von Korff M, Rufener C (2015). Data warrior: an open-source program for chemistry aware data visualization and analysis. J Chem Inf Model.

[CR38] Sarmah S, Marrs JA (2016) Zebrafish as a vertebrate model system to evaluate effects of environmental toxicants on cardiac development and function. Int J Mol Sci. 10.3390/ijms17122123 10.3390/ijms17122123 PMC5187923 27999267

[CR39] Shi Z, Chen Y, Lu C (2018). Resolving neuroinflammation, the therapeutic potential of the anti-malaria drug family of artemisinin. Pharmacol Res.

[CR40] Stevens JL, Baker TK (2009). The future of drug safety testing: expanding the view and narrowing the focus. Drug Discov Today.

[CR41] Stewart A, Huang J, Fisher RA (2012). RGS proteins in heart: brakes on the vagus. Front Physiol.

[CR42] Todorova VK, Beggs ML, Delongchamp RR (2012). Transcriptome profiling of peripheral blood cells identifies potential biomarkers for doxorubicin cardiotoxicity in a rat model. PLoS ONE.

[CR43] Wang W, MacKinnon R (2017) Cryo-EM structure of the open human ether-a-go-go-Related K(+) channel hERG. Cell 169(3):422–430 e10 10.1016/j.cell.2017.03.048 10.1016/j.cell.2017.03.048 PMC5484391 28431243

[CR44] Yan G, Dawood M, Bockers M (2020). Multiple modes of cell death in neuroendocrine tumors induced by artesunate. Phytomedicine.

[CR45] Yin JY, Wang HM, Wang QJ (2014). Subchronic toxicological study of two artemisinin derivatives in dogs. PLoS ONE.

[CR46] Yoon CS, Kim HK, Mishchenko NP, et al. (2018) Spinochrome D attenuates doxorubicin-induced cardiomyocyte death via improving glutathione metabolism and attenuating oxidative stress. Mar Drugs. 10.3390/md17010002 10.3390/md17010002 PMC6356724 30577438

[CR47] Zhang S, Meng T, Liu J, Zhang X, Zhang J (2015). Cardiac protective effects of dexrazoxane on animal cardiotoxicity model induced by anthracycline combined with trastuzumab is associated with upregulation of calpain-2. Med (Baltim).

[CR48] Zheng C, Shan L, Tong P, Efferth T (2020). Cardiotoxicity and cardioprotection by artesunate in larval zebrafish. Dose Response.

